# Marginal Sealing Durability of Two Contemporary Self-Etch Adhesives

**DOI:** 10.5402/2012/204813

**Published:** 2012-04-29

**Authors:** Maryam Khoroushi, Mahsa Mansoori

**Affiliations:** ^1^Dental Materials Research Center and Department of Operative Dentistry, School of Dentistry, Isfahan University of Medical Sciences, Hezar Jerib Street, Isfahan 81746-73461, Iran; ^2^Torabinejad Dental Research Center, School of Dentistry, Isfahan University of Medical Sciences, Hezar Jerib Street, Isfahan 81746-73461, Iran; ^3^Department of Pediatric Dentistry, Tehran University of Medical Sciences, Tehran 14399-55991, Iran

## Abstract

*Introduction*. Sealing abilities of two self-etch adhesives were evaluated after two aging processes: storage in water and thermocycling. *Materials and Methods*. Cl V cavities were prepared on the buccal and lingual aspects of 48 human premolars, with cervical margins 1 mm below the CEJ. Clearfil Protect Bond (CPB) and BeautiBond (BB) (two-step and one-step self-etch adhesives, resp.) were applied, each to half of the cavities and restored with composite resin. Each group was randomly subdivided into 4 subgroups (*n* = 12) and evaluated for dye penetration after 24 hours, after 3000 thermocycling rounds, after a 6-month water storage, and after 3000 thermocycling rounds plus 6-month water storage, respectively. Data was analyzed using SPSS 11.5 and Kruskal-Wallis and Mann-Whitney *U* tests (*α* = 0.05). *Results*. There were no significant differences in enamel and dentin microleakage between the adhesives (*P* = 0.683; *P* = 0.154). Furthermore, no significant differences were observed in enamel microleakage of each one of CPB and BB (*P* = 0.061 and *P* = 0.318, resp.). However, significant decrease was observed in subgroups 3 and 4 (*P* = 0.001) for CPB dentinal margins. *Conclusion*. In this study, limited aging procedures had no influence on marginal integrity of composite resin restorations bonded with self-etch adhesives of CPB and BB. Furthermore, CPB dentinal sealing improved after aging.

## 1. Introduction

Recurrent caries is one of the most common problems after tooth restoration, especially with tooth-colored resin materials. A large number of studies have indicated that microleakage is the most important reason for recurrent caries and pulp inflammation and necrosis [1]. Initial studies claimed that the restorative materials themselves are the direct cause of pulp irritation; however, subsequent studies showed that these irritations are the result of microleakage [2]. In the case of tooth-colored restorations some factors are associated with microleakage at margins, which include bond strength of the adhesive to tooth structures, residual stresses as a result of polymerization shrinkage of composite resin, differences in the thermal expansion coefficients of enamel and dentin with the restorative materials, and mechanical stresses to the restoration during occlusion [3].

In recent years the etching step of the substrate has been incorporated into the priming and bonding steps, leading to the self-etch strategy, which has been welcomed by dental practitioners due to its ease of application. This new trend in the newer generations of these materials has enabled etching, priming, and bonding steps in one single step [4, 5]. Based on the results of studies available now, although both etch-and-rinse and self-etch adhesives exhibit acceptable laboratory and clinical results, the quality and efficacy of each adhesive system depend, to a great degree, on its formulation and is highly material dependent [5]. Meanwhile, not only do one-step simplified systems produce a weaker bond, but also their bond durability is less predictable clinically [6]. The morphologic appearance of the hybrid layer produced by self-etch adhesives is, to a great degree, dependent on the type and composition of functional monomers and their ability to demineralize dentin. In this context, these adhesives are divided into strong (pH ≤ 1), intermediate (pH *≈* 1.5) and mild (pH ≥ 2) self-etch adhesives. In addition, newer adhesives with lower acid strength, referred to as ultramild, are available [7, 8].

Furthermore, formulations of bonding agents have undergone great changes in recent decade [7]. The most important challenge for dental adhesives is the production of a durable bond between two materials with different structures [9]. Modern adhesives are very technique sensitive, and any minor mistakes in their use might give rise to disturbances in the bonding process. Therefore, an adhesive with a durable bond, easy application, and low technique sensitivity is still a clinical necessity. Consequently, research is still underway by manufacturers and they sometimes introduce newer products with claims of better bonding properties [5].

The majority of newer formulations of adhesive systems contain fluoride and various antibacterial agents. In this context, it appears that long-term research studies are necessary to evaluate various adhesive systems. Most of the common bonding systems have exhibited high initial bond strength values after 24 hours of storage in water. However, some laboratory studies have shown that bond strength decreases after several months of storage in water [10]. Hence, it is necessary to evaluate the bond longevity and marginal integrity of various adhesive systems, especially those with newer formulations containing fluoride and antibacterial agents or with new and different functional molecules.

Although laboratory evaluations and in vitro studies cannot exactly simulate the oral cavity conditions, such as the chemical environment, moisture, and the stresses inflicted on the teeth and restorations, they can, to some extent, simulate the oral cavity environment through aging procedures of teeth and/or restorations. As a result, it appears that the results of such studies are, as far as possible, similar to the outcomes obtained in clinical situations under complex occurrences in the oral cavity [11–13].

Clearfil Protect Bond (CPB) is a type of two-step self-etch adhesive, that contains fluoride and a functional monomer as an antibacterial agent. In addition, both the primer and the bonding agent have 10-MDP in their chemical compositions [14]. According to some studies, aging processes such as storage in water and thermocycling do not influence the bond strength of this adhesive [4, 11]. One study has reported an increase in the bond strength of CPB after storage in water for one year [4]. BeautiBond (BB) is a type of new one-step self-etch adhesive that contains phosphonic and carboxylic agents for chemical bond to enamel and dentin, with no decrease in bond strength after storage in water based on a claim by the manufacturer [15]. Nevertheless, no studies have been carried out on its wall integrity under normal conditions and aging procedures. Therefore, the aim of the present study was to evaluate initial and delayed microleakage of these two self-etch adhesive systems after storage in water and application of thermocycling procedures.

## 2. Materials and Methods

In this experimental laboratory study, 48 sound human premolars were used; the teeth did not have any carious lesions, restorations, abrasions, and cracks and had been extracted less than 3 months before being used for the purpose of the study. The teeth were cleansed using a brush after removing any periodontal fibers and bone remnants. Then, they were stored in 0.2% thymol solution at 4°C. Twenty-four hours before initiation of the study, the teeth were retrieved from the thymol solution and stored in distilled water at 37°C before being prepared. Class V cavities were prepared on the buccal and lingual surfaces of the teeth, which measured 4 mm mesiodistally, 2 mm occluso-gingivally, and 1.5 mm in depth. A total of 12 diamond fissure burs (D & Z, Hilzingen, Germany) with a diameter of 1 mm were used for cavity preparations; one new bur was used for every four preparation procedures. The occlusal and gingival cavity margins were placed on the enamel and 1 mm apical to CEJ, respectively. All the cavity preparations were carried out using one handpiece at 120,000 rpm under air and water spray. Half of the cavities were restored with the one-step self-etch BB adhesive, and the other half were restored with the one-step self-etch CPB adhesive in two groups according to manufacturers instructions (Table 1). All the cavities were restored with the A3 shade of APX composite resin (APX, Kuraray, Tokyo, Japan), using the incremental technique.

Subsequent to restoration of the cavities, all the teeth were stored in an incubator (Behdad, Tehran, Iran) for 24 hours in distilled water at 37°C to decrease stresses resulting from polymerization. Then the restorations were polished using flame-shaped polishing burs, polishing disks (3M ESPE, St. Paul, MN, USA), and cup-shaped polishing rubbers from coarse to fine. Then, each group (*n* = 24) was randomly divided into 4 subgroups of 6. The samples in subgroups 1, 2, 3, and 4 were prepared for microleakage test after 24 hours, after 3000 rounds of thermocycling, after 6 months of storage in distilled water in an incubator, and after 3000 rounds of thermocycling and storage in water in an incubator for 6 months, respectively. In order to evaluate microleakage, the apexes of all the teeth were sealed with sticky wax and all tooth surfaces were coated with 3 layers of nail varnish except for a 1 mm periphery of the cavities. All the samples were placed in an incubator for 24 hours in 2% basic fuchsin solution at 37°C. Each sample was mounted in self-curing acrylic resin to facilitate cutting of the sample. The samples were cut in a buccolingual direction parallel to tooth long axis using a cutting machine and diamond disk (Lemgo, Germany). Then, each specimen was graded for dye penetration under a stereomicroscope (MBC-10, St. Petersburg, Russia) at ×16 and ×32. Dye penetration was graded as follows:

(0) no dye penetration,
(1) dye penetration up to 1/3 of the cavity depth,
(2) dye penetration up to 2/3 of the cavity depth,
(3) dye penetration more than 1/3 of the cavity depth toward the pulp.

Data was analyzed using SPSS 11.5 statistical software with Kruskal-Wallis and Mann-Whitney tests at a 95% confidence interval.

## 3. Results

**Figure 1:**
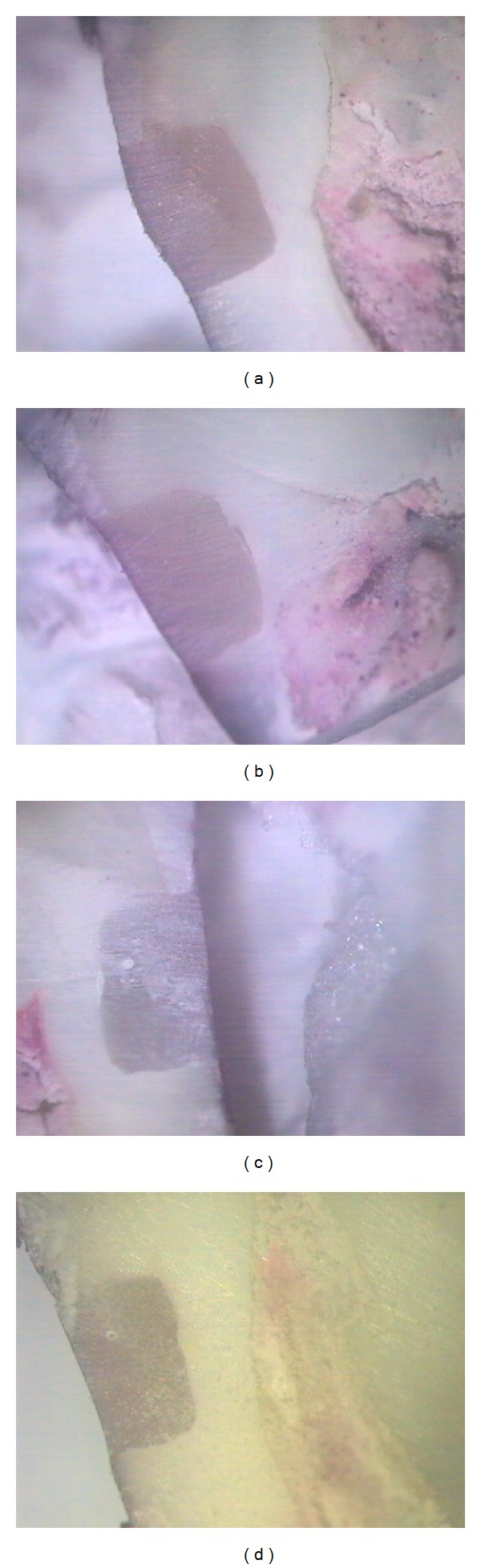
(a) Enamel and dentin margins of a specimen belonging to group CPB1: CPB after 24 h (×16). (b) Enamel and dentin margins of a specimen belonging to group CPB2: CPB after 3000 thermal cycles (×16). (c) Enamel and dentin margins of a specimen belonging to group CPB3: CPB after 6 months of water storage (×16). (d) Enamel and dentin margins of a specimen belonging to group CPB4: CPB after 3000 thermal cycles plus 6 months of water storage (×16).

**Figure 2:**
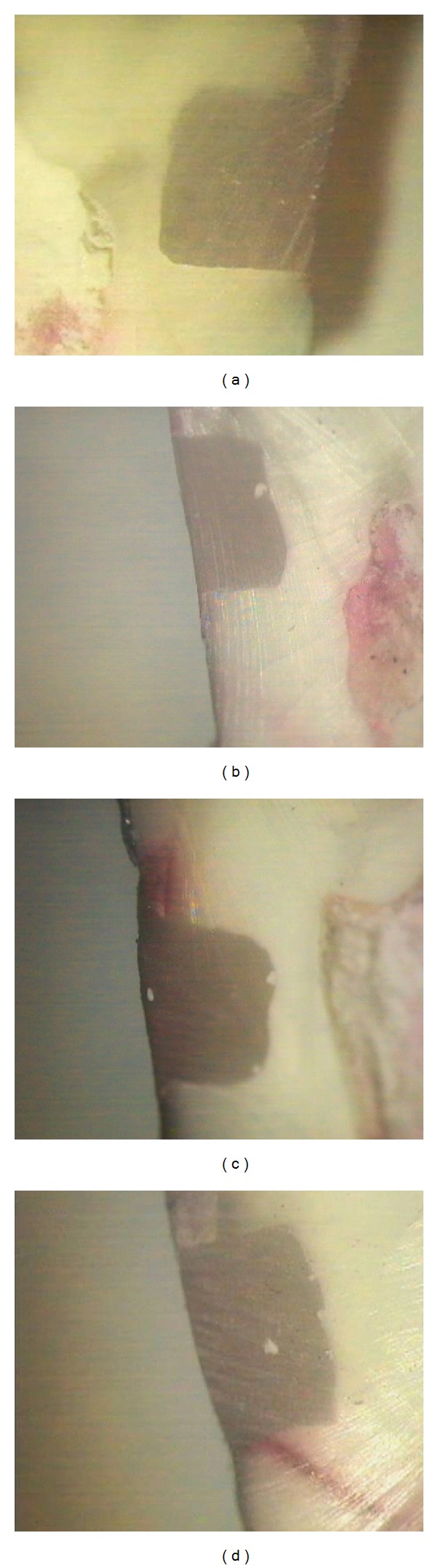
(a) Enamel and dentin margins of a specimen belonging to group BB1: BB after 24 h (×16). (b) Enamel and Dentin margins of a specimen belonging to group BB2: BB after 3000 thermal cycles (×16). (c) Enamel and dentin margins of a specimen belonging to group BB3: BB after 6 months of water storage (×16). (d) Enamel and dentin margins of a specimen belonging to group BB4: BB after 3000 thermal cycles plus 6 months of water storage (×16).

Tables 2, 3 and 4 summarize the obtained microleakage scores of the eight studied groups. The Kruskal-Wallis test did not reveal any significant differences in the means of microleakage between the two adhesives under study (*P* = 0.681). There were no significant differences in the enamel microleakage of any of the subgroups with the two adhesives under study (*P* = 0.318, and *P* = 0.061, resp.). In addition, no significant differences were observed in the microleakage of the two adhesives under study (*P* = 0.154). However, with the CPB, there were significant differences in the dentin microleakage of subgroup 1 and subgroups 3 and 4 (*P* = 0.001), with a decrease in the dentin microleakage of the CPB adhesive after storage in water (Table 4; Figures 1 and 2) and less marginal sealability for BB in coparison to CPB (Figure 2).

Two-by-two comparison of corresponding groups with Mann-Whitney test showed significant differences in the enamel margins of subgroups 3 (*P* = 0.012), with significantly less enamel microleakage with CPB after 6 months compared to BB (Table 4). 

In relation to comparison of the two substrates, no significant differences were observed between the dentin and enamel microleakage with the two CPB and BB adhesives (*P* = 0.826 and *P* = 0.09, resp.) (Table 4).

## 4. Discussion

Water storage is one of the most commonly used in vitro techniques to predict the behavior of resin restorations [11]. In addition, application of thermocycling procedures is an accepted technique for in vitro evaluation of restorations [13].

According to the results of the present study, none of the two adhesives produced a complete marginal seal in dentin and enamel substrates. Previous studies have shown that application of different adhesives does not result in complete elimination of marginal microleakage [16–19]. While for the etch-and-rinse adhesives the bond failure is a result of hydrolysis and enzymatic degradation in the collagen fibers and in the polymerized resin matrix within the hybrid layer, in the case of self-etch adhesive systems the bond failure mechanism and loss of marginal seal are relatively unknown. Mild self-etch adhesives demineralize dentin to some extent. Initially, it was believed that adequate penetration of resin will not occur with these adhesives because with the application of these materials the two etching and priming steps occur simultaneously in addition to the relative etching of dentin [11]. However, two studies reported nanoleakage at the hybrid layer of these adhesives and attributed it to incomplete resin penetration [20, 21]. Another study attributed this phenomenon to the continued etching in these adhesives [22]. Other reasons were also reported later, including the presence of acidic functional monomers that significantly increase the hydrophilicity of the adhesive layer [11]. 

 Despite great advances in adhesive systems, the tooth-restoration interface is still the weakest part of tooth-colored restorations [23]. Clinically, margins placed more apical to CEJ pose problems in the control of moisture and bonding to dentin because dentin has a “nonhomogeneous” structure and other conditions such as the presence of hydroxyapatite, collagen, the smear layer, dentinal tubules and tubular fluid should be considered to form a proper bond with dentin [24].

In the present study two types of one-step and two-step self-etch adhesives were used to evaluate the effect of aging on marginal integrity. Microleakage of two-step self-etch adhesives after a six-month period in groups 3 and 4 exhibited less decrease in the dentinal margin. Bond durability is an important factor in maintaining the integrity of restorations bonded with the use of adhesives [11]. Some problems have been reported with the use of one-step self-etch adhesives, including inadequate polymerization, water sorption, nanoleakage of water or enzymes, and presence of bubbles leading to osmosis and phase separation [11]. These adhesives are hydrophilic after polymerization and tend to absorb more water. Therefore, they can act as a penetrable and semipermeable material to allow water to pass through, which results in a decrease in the mechanical properties of the resin and bond durability [25, 26]. In the present study, no significant differences were observed between microleakage of CPB and BB adhesives. It appears that BB has a more advanced formulation since it has separate and specific functional molecules to bond to enamel and dentin compared to other more recognized functional molecules and its initial dentinal and enamel seal is comparable to two-step self-etch bonding systems.

In the present study, no significant differences were observed in microleakage between groups 1 and 2 with the use of both CPB and BB adhesive systems at enamel and dentin walls. In other words, the use of thermocycling procedures in the range of 3000 rounds did not influence marginal seal of these two adhesives. In a study carried out by Rossomando and wendt, too, thermocycling or lack of it did not have any effect on microleakage of composite resin restorations [27]. Moreover, In this study, the samples in group 2 underwent a 3000-round thermocycling procedure after restoration. In this technique the samples are subjected to thermocycling procedures to simulate thermal changes in the oral cavity. However, a wide range of temperatures and time intervals have been used for these procedures in different studies. Therefore, no specific standard exists for the use of this technique in microleakage studies and other in vitro studies and there are conflicting results and controversy over the interpretation of results of different studies [13]. Nevertheless, based on the results of the present study, thermocycling had no effect on the enamel and dentin margin microleakage of BB. In contrast, Rosales-Leal evaluated microleakage of Cl V cavities after application of a number of etch-and-rinse (Prime & Bond NT, XP Bond, Scotchbond 1 XT, Syntac) and self-etch (Xeno III, i-Bond, Clearfil SE bond) adhesive systems before and after thermocycling procedures. Thermocycling did not decrease gingival seal in all the bonding systems under study except for SBX and XPB [18]. Based on the results, although thermocycling did not have any effect on enamel seal, its effect on dentinal seal was different depending on the adhesive materials.

In this study in group 3, in which the restorations were stored in water for 6 months, no differences were observed in the microleakage of BB adhesive system at enamel and dentin margins. However, microleakage at margins in the CPB adhesive system showed a significant decrease at dentinal margin. Clearfil Protect Bond (CPB) is a type of two-step self-etch adhesive, that contains fluoride and MDPB functional monomer as an antibacterial agent. In addition, both the primer and the bonding agent have 10-MDP in their chemical compositions [14]. Moreover, Nakajima et al. reported that the bond produced by adhesives with high fluoride content improves after 6 months of storage in water. They argued that fluoride increases dentin strength and stability, increasing bond durability [28]. This adhesive has MDPB monomer (12-methacryloyloxydodecylpyridinium bromide) in its primer, which ruins the bacteria present in the cavity and decreases bacterial growth and proliferation under the restoration [14]. In addition, recently, microscopic studies have revealed a zone under the hybrid layer of self-etch adhesives, which is resistant to acids named “acid-base resistant zone” (ABRZ). This layer is completely different from the layer that forms under fluoride-releasing materials such as GI cements. Although ABRZ is also produced under the adhesives that do not release fluoride, it has minimal thickness in such cases. It has been reported that ABRZ prevents recurrent caries; in addition, its morphology depends, to a great degree, on the materials it is composed of [29]. Shinohara et al. evaluated various self-etch primers and reported that ABRZs produced by fluoride-releasing adhesives are thicker than those produced by other adhesives [30]. It is probable that formation of ABRZ is the result of penetration of adhesive monomers into relatively demineralized dentin. In addition, it is possible that release of fluoride, too, is involved in the formation of ABRZ. When self-etch adhesives, such as CPB, are used, dentin becomes partially demineralized in such a way that some hydroxyapatite crystals remain in the hybrid layer and bond to 10-MDP monomer, producing a kind of salt, which is relatively insoluble [29]. Since ABRZ is mechanically, biologically, and chemically more resistant than normal dentin, it is referred to as “super dentin” [29].

On the other hand, the favorable chemical bond seen with some self-etch adhesives is due to the presence of three chemical ingredients of 10-MDP, 4-META, and Phenyl-P, which have phosphate and carboxylic groups and can form ionic bonds with the calcium within hydroxyapatite. However, of the three chemical agents, the bond produced by 10-MDP is stronger and more durable than those formed by the two other agents [6]. CPB is a 10-MDP-based adhesive and contains this chemical agent in both its primer and adhesive structure. Therefore, the results of the present study in relation to this adhesive in groups 3 and 4 appear to be rational [6–8].

In the present study initial and delayed microleakage of BB at both enamel and dentin substrates was not generally greater than that with the two-step adhesive and did not increase after aging procedures. It has been reported that an important factor effective in the bond strength and possibly the seal of self-etch adhesives is the type of the smear layer [5, 6]. Depending on the technique and the tool used for cavity and specimen preparation, the thickness, density and adhesion properties of the smear layer to tooth structure are different. Self-etch adhesives do not remove the smear layer; rather they interact with it. Therefore, the acidic potential of the adhesive and the extent to which the mineral content of a dense and thick smear layer is buffered are affected. This phenomenon might result in a weaker interaction of the adhesive with the substrate. It appears that the weaker the adhesive is the greater the smear layer interferes with the bonding process. Extrafine diamond burs have been recommended to finish cavities restored with the use of mild and ultramild self-etch adhesives [6], the effect of which on microleakage of different adhesives requires further evaluations.

Another important consideration is the fact that BB is a mild one-step self-etch adhesive without HEMA. Such adhesives are less hydrophilic and produce a more durable bond with the substrate. At present, hydrolysis is considered as an important factor involved in the bond failure of these resin materials [5, 9, 12]. Studies have shown that more hydrophilic adhesives absorb more water compared to hydrophobic resins. Water sorption softens the polymer and reduces its strength and hardness; therefore, the dentin-resin bond durability is compromised and bond strength decreases with time [31]. On the other hand, it should be pointed out that HEMA makes the adhesive more susceptible to phase separation; therefore, when the solvent (ethanol or acetone) begins to vaporize, the equilibrium between the monomer and the solvent in the chemical composition of the adhesive is disturbed and water is separated from other components. When the adhesive is cured, these water droplets appear as blisters within and on the adhesive layer and compromise the bond. Therefore, the application of strong and continuous air currents on the adhesive layer is usually recommended with HEMA-free adhesives before polymerization so that these water droplets would be removed. However, it is not clear how successful such a measure is from a clinical viewpoint [6]. The manufacturer of BB, which is an HEMA-free adhesive, recommends a 3-second application of air with mild pressure and a 2-second application of a strong air current. Regarding the results in groups 3 and 4 of the present study, it appears that the use of HEMA-containing adhesive, CPB, is preferable. At present, the presence of controlled amounts of HEMA, to establish an equilibrium between the monomer and water, is preferred to HEMA-free and HEMA-rich adhesives [6, 31]. Finally, since the majority of microleakage studies have been carried out in laboratory conditions and no long-term and diverse aging procedures have been carried out to evaluate marginal seal, it is suggested that more comprehensive evaluations be carried out. However, such evaluations do not eliminate the need for clinical studies.

## 5. Conclusion

Under the conditions of the present study and regarding its limitations, limited aging procedures did not have any effect on the seal of enamel and dentin margins of composite restorations with the use of BB and CPB self-etch adhesives. The dentin seal of CPB increased after 6 months of storage in water and thermocycling. Further studies are recommended.

## Figures and Tables

**Table 1:** Adhesive resins used, their compositions, and their mode of application according to the manufacturers instructions.

Adhesive resin and manufacturer	Materials compositions	Manufacturers directions
BB (one-step self-etch adhesive, Shofu company)	Bis-GMA, TEGDMA, phosphonic acid monomer, carboxylic acid monomer, acetone, and water	Leave bonding for 10 sec. Air dry with gentle air for about 3 sec, and then dry with stronger air until a thin and uniform bonding layer is obtained. Light-cure for 10 sec with a dental curing light unit, Halogen (irradiation wave length: 400–500 nm, light intensity: >500 mW/s)

CPB (two-step, self-etch adhesive, pH = 2.5, Kuraray, Osaka, Japan)	Primer: water, MDPB, HEMA, dimethacrylates, and photoinitiator Bonding resin: MDP, HEMA, dimethacrylates, silanted, colloidal silica and sodium fluoride	Apply primer gently on the surface and leave undisturbed for 20 seconds. Gently air blow. Apply bond. Air-thin and light-cure for 10 seconds

HEMA: hydroxyl ethyl methacrylate; MDPB: methacryloyloxydodecylpyridinium bromide; MDP: methacryloxydecyl dihydrogen phosphate; CPB: Clearfil Protect Bond; BB: BeautiBond.

**Table 2:** Microleakage distribution in enamel margins in the study groups.

Adhesive resin	Groups numbers and definitions	Scores				
		0	1	2	3	Total
CPB	(1) 24 h	8	3	1	0	12
		66.7%	25.0%	8.3%	0.0%	100%
	(2) T	7	2	0	3	12
		58.3%	16.7%	0.0%	25.0%	100%
	(3) Ws	10	2	0	0	12
		83.3%	16.7%	0.0%	0.0%	100%
	(4) Ws & T	6	5	0	1	12
		50.0%	41.7%	0.0%	8.3%	100%

BB	(1) 24 h	7	3	2	0	12
		58.3%	25.0%	16.7%	0.0%	100%
	(2) T	10	1	1	0	12
		83.3%	8.3%	8.3%	0.0%	100%
	(3) Ws	3	8	1	0	12
		25.0%	66.7%	8.3%	0.0%	100%
	(4) Ws & T	8	4	0	0	12
		66.7%	33.3%	0.0%	0.0%	100%

CPB: Clearfil Protect Bond; BB: BeautiBond; WS: water stored; T: thermocycled.

**Table 3:** Microleakage distribution in dentin margins in the study groups.

Adhesive resin	Groups numbers and definitions	Scores				
		0	1	2	3	Total
CPB	(1) 24 h	4	6	2	0	12
		33.3%	50.0%	16.7%	0.0%	100%
	(2) T	6	2	1	3	12
		50.0%	16.7%	8.3%	25.0%	100%
	(3) Ws	12	0	0	0	12
		100.0%	0.0%	0.0%	0.0%	100%
	(4) Ws & T	12	0	0	0	12
		100.0%	0.0%	0.0%	0.0%	100%

BB	(1) 24 h	10	1	1	0	12
		83.3%	8.3%	8.3%	0.0%	100%
	(2) T	11	0	0	1	12
		91.7%	0.0%	0.0%	8.3%	100%
	(3) Ws	9	2	1	0	12
		75.0%	16.7%	8.3%	0.0%	100%
	(4) Ws & T	10	1	1	0	12
		83.3%	8.3%	8.3%	0.0%	100%

CPB: Clearfil Protect Bond; BB: BeautiBond; WS: water stored; T: thermocycled.

**Table 4:** Mean rank of dye penetration in the different study groups.

Substrate	Groups numbers and definitions	Mean rank	
		CPB	BB
Enamel	(1) 24 h	23.71^Aa^	25.33^Aa^
	(2) Thermocycled	27.21^Aa^	19^Aa^
	(3) Water stored	19.50^Aa^	31.83^Ba^
	(4) Thermocycled and water stored	27.50^Aa^	21.83^Aa^

Dentin	(1) 24 h	32.42^Aa^	24.46^Aa^
	(2) Thermocycled	30.58^Aa^	22.79^Aa^
	(3) Water stored	17.50^Ab^	24.46^Aa^
	(4) Thermocycled and water stored	17.50^Ab^	26.29^Aa^

Means followed by different letters show statistical differences (*α* = 0.05). Uppercase letters: comparison of each procedure for each adhesive (row). Lowercase letters: comparison of adhesives at each procedure (column). CPB: Clearfil Protect Bond; BB: BeautiBond; WS: water stored; T: thermocycled.
